# Supplementary material for: Individualized Pooled CRISPR/Cas9 Screenings Identify CDK2 as a Druggable Vulnerability in a Canine Mammary Carcinoma Patient
Source: Vet Sci. 2025 Feb 18;12(2):183. doi: 10.3390/vetsci12020183 (PMC11861728; doi:10.3390/vetsci12020183)
Supplement: Supplementary file 1 [file vetsci-12-00183-s001.zip › S3, S5, S7-S9.pdf]

Table S1. All 1269 genes targeted by the custom canine CRISPR/Cas9 sub-library CP1737 and sgRNAs counts (plasmid DNA and day 0 and day 40 for both organoid lines)

This data is available in a separate downloadable Excel file (Supplementary Table S1).

Table S2. All 834 genes targeted by the custom canine CRISPR/Cas9 sub-library CP1736 and sgRNAs counts (plasmid DNA and day 0 and day 40 for both organoid lines)

This data is available in a separate downloadable Excel file (Supplementary Table S2)

Table S3. All hits for the epigenome screen.

Hits represented have at least 4 sgRNA out of 6 taken into account for analysis. In bold are represented the hits significant for ORG-63-C (LFC < -1 and p-value < 0,05) but not significant for ORG-63-N (LFC > -1 or p-value > 0,05). Fdr= false discovery rate

Gene	p-value_N	fdr_N	lfc_N	p-value_C	fdr_C	lfc_C
**SPEN**	0.91413	0.999998	1.531	0.024543	0.56797	-2.1261
**VPS72**	0.8979	0.999998	0.4675	0.016025	0.535589	-2.577
**PLOD2**	0.8599	0.999998	1.6872	0.01308	0.52067	-3.3332
**NCOR1**	0.76821	0.999998	0.25754	0.021244	0.535589	-1.8888
**SRSF2**	0.68095	0.999998	0.46804	0.015419	0.535589	-2.9553
**HDAC1**	0.6639	0.999998	0.50999	0.045327	0.706395	-1.6468
**ACTL6A**	0.60347	0.999998	0.48882	0.0094057	0.450198	-1.6701
**TBL1X**	0.60168	0.999998	-0.44383	0.046566	0.709117	-2.3178
**BAZ1A**	0.59139	0.999998	-0.22595	0.014092	0.535589	-2.3712
**NOC2L**	0.58843	0.999998	-0.46931	0.018451	0.535589	-2.2209
**TAF8**	0.58046	0.999998	-0.35214	0.018177	0.535589	-1.5937
**TFDP1**	0.46931	0.999998	0.37269	0.033807	0.618612	-2.0121
**CBX5**	0.45174	0.999998	0.40584	0.039584	0.649156	-1.4734
**SMARCA5**	0.44409	0.999998	0.87841	0.013878	0.535589	-2.9798
**SRRM1**	0.43516	0.999998	-0.43184	0.019006	0.535589	-1.8592
**COPS5**	0.43285	0.999998	0.75487	0.015724	0.535589	-3.1107
**NAP1L1**	0.40623	0.999998	-0.30089	0.024866	0.56797	-2.7199
**HASPIN**	0.40398	0.999998	-0.59461	0.0077781	0.410431	-3.1853
**RCC1**	0.39469	0.999998	0.23547	0.01764	0.535589	-1.5031
**OIP5**	0.38418	0.999998	-0.72621	0.018399	0.535589	-2.2176
**SRSF5**	0.34631	0.999998	-0.85432	0.04839	0.716825	-1.9515
**MIS18BP1**	0.34095	0.999998	-1.0051	0.019045	0.535589	-2.8395
**RBPMS**	0.33435	0.999998	-0.93472	0.047674	0.716379	-1.9591
**SETD1A**	0.32603	0.999998	-0.64911	0.020014	0.535589	-1.4904
**CDK2**	0.31995	0.999998	-0.74951	0.024024	0.567811	-1.3541
**USP7**	0.31791	0.999998	-1.0529	0.027406	0.568722	-1.8601
**SUZ12**	0.31684	0.999998	-0.45366	0.019363	0.535589	-3.0567
**DHX38**	0.31248	0.999998	-0.62685	0.025534	0.568722	-2.5343
**KDM8**	0.30885	0.999998	-0.88315	0.0074727	0.403701	-2.061
**OTUB1**	0.30217	0.999998	-0.90419	0.017099	0.535589	-1.6885
**GTF3C4**	0.29667	0.999998	-0.10293	0.048652	0.716825	-2.0456
**CENPA**	0.2798	0.999998	-0.89153	0.0084414	0.425633	-2.9489
**ZZZ3**	0.26212	0.999998	-0.83309	0.017243	0.535589	-3.2132

**CHEK1**	0.25733	0.999998	1.0502	0.02631	0.568722	-2.5633
**DDX17**	0.24814	0.999998	0.56402	0.031922	0.610493	-2.85
**SNAPC4**	0.24499	0.999998	-0.97962	0.021048	0.535589	-2.8467
ABT1	0.2041	0.999998	-1.3441	0.040989	0.65961	-2.9987
MTA2	0.19434	0.999998	-1.0678	0.036565	0.638614	-2.2856
RAP2C	0.18985	0.999998	-1.0954	0.015659	0.535589	-2.3211
**MED30**	0.17747	0.999998	-0.62673	8.51E-05	0.064356	-2.7196
CXXC1	0.17486	0.999998	-1.5147	0.0059803	0.38855	-2.968
SPRTN	0.16608	0.999998	-1.4722	0.04407	0.699266	-1.7489
SMC1A	0.16064	0.999998	-1.4101	0.021139	0.535589	-2.2505
GTF3C3	0.15084	0.999998	-1.1418	0.037512	0.638614	-1.9039
**PCNA**	0.14632	0.999998	-0.68459	0.012683	0.51547	-3.0623
BMI1	0.1407	0.999998	-1.5576	0.033654	0.618612	-2.3752
SRSF11	0.13836	0.999998	-1.3455	0.0055614	0.38855	-1.0892
GTF2A1	0.12769	0.999998	-1.7166	0.027279	0.568722	-2.7225
**ECD**	0.12707	0.999998	-0.28612	0.026677	0.568722	-3.0257
PRMT5	0.11933	0.999998	-1.629	0.018478	0.535589	-2.0565
**RSF1**	0.11897	0.999998	-0.7464	0.032301	0.610493	-2.8841
**UBE2A**	0.11589	0.999998	-0.96186	0.010972	0.47877	-1.3365
**GTF2B**	0.1149	0.999998	-0.77955	0.023854	0.567811	-2.7909
PPP1CB	0.10852	0.999998	-1.5541	0.0051643	0.38855	-2.9689
SMC4	0.1079	0.999998	-1.7961	0.026494	0.568722	-1.9127
**SMC2**	0.10679	0.999998	-0.38099	0.0066392	0.38855	-3.1905
ADRM1	0.10272	0.999998	-1.3305	0.020463	0.535589	-1.2524
NFYA	0.098148	0.998646	-1.5585	0.037996	0.638614	-2.5381
**RUVBL1**	0.096328	0.993497	0.44603	0.0039294	0.38855	-3.0927
PELP1	0.09467	0.992292	-1.5954	0.0036501	0.38855	-2.5978
NELFB	0.094107	0.992292	-1.3899	0.024499	0.56797	-2.6692
POLR1B	0.088199	0.95753	-1.7391	0.039768	0.649156	-2.5336
JMJD6	0.078193	0.953875	-1.3838	0.0044443	0.38855	-2.1815
SF1	0.074323	0.952761	-1.7692	0.0041214	0.38855	-2.5906
MYC	0.07054	0.930549	-1.273	0.045453	0.706395	-2.1414
TPR	0.069274	0.9192	-1.6911	0.042953	0.686341	-1.1981
**AURKB**	0.068751	0.917618	-0.94293	0.0055614	0.38855	-3.0714
PHB	0.066682	0.900607	-1.5405	0.018395	0.535589	-2.4287
UHRF1	0.066132	0.900607	-1.9241	0.037128	0.638614	-2.8416
MNAT1	0.053635	0.785149	-1.822	0.028632	0.574915	-2.5665
YY1	0.050751	0.762606	-1.3351	0.025032	0.56797	-2.2115
CTCF	0.049952	0.760099	-1.2604	0.0026247	0.358474	-2.5464
TICRR	0.04969	0.760099	-2.1686	0.032825	0.610493	-2.4729
POLR2J	0.049477	0.760099	-1.6569	0.017788	0.535589	-2.641
CCAR1	0.049415	0.760099	-1.7598	0.00050399	0.228713	-3.5672
NELFCD	0.048787	0.760099	-1.9668	0.037725	0.638614	-2.4954
UXT	0.048582	0.760099	-1.0739	0.0046014	0.38855	-2.9286
SLBP	0.048504	0.760099	-2.0338	0.020956	0.535589	-3.0779
POLR2F	0.04805	0.760099	-1.9746	0.036403	0.638614	-2.2638
HELLS	0.04719	0.760099	-1.9562	0.008437	0.425633	-2.3175
POLR2G	0.047081	0.760099	-2.2205	0.0026858	0.358474	-3.1486
ELOB	0.040719	0.721805	-1.5608	0.0096064	0.450198	-2.7374
POLR2H	0.04013	0.716964	-1.3053	0.049285	0.721463	-2.291
COMMD3	0.03941	0.716964	-0.08309	0.020489	0.535589	-2.1333
MED22	0.036927	0.708003	-1.5105	0.0047541	0.38855	-2.2366
BUD23	0.036451	0.706905	-1.9248	0.040989	0.65961	-2.4385
PRPF6	0.035827	0.700794	-1.8364	0.014638	0.535589	-3.0694
AIFM1	0.034998	0.700794	-2.2822	0.004453	0.38855	-3.6003

POLR2I	0.033152	0.690117	-2.4045	0.0021622	0.342904	-3.8492
TERF1	0.030827	0.659864	-2.6102	0.03934	0.649156	-2.5534
CENPC	0.029897	0.652275	-1.492	0.010614	0.472238	-2.7812
HMGB1	0.028702	0.638468	-2.3124	0.0211	0.535589	-1.197
GTF3C1	0.028156	0.638468	-1.8925	0.012722	0.51547	-0.3519
POLR2A	0.028038	0.638468	-1.6454	0.00044727	0.228713	-4.0812
PAF1	0.02734	0.638468	-1.2253	0.01527	0.535589	-3.2934
TAF3	0.027305	0.638468	-2.2149	0.0041432	0.38855	-2.216
TAF2	0.026389	0.638468	-1.7142	0.0049112	0.38855	-2.9102
MBD2	0.023243	0.612553	-1.0074	0.0022494	0.342904	-3.0525
ESPL1	0.020978	0.595483	-2.2858	1.09E-05	0.024752	-5.7319
DNAJC2	0.020961	0.595483	-2.4426	0.0099206	0.450198	-3.296
CHAF1B	0.018403	0.579964	-1.866	0.029902	0.595145	-2.8709
ASH2L	0.018316	0.579964	-1.7625	0.034784	0.631406	-2.5981
POLR1C	0.017806	0.579964	-2.3391	0.005653	0.38855	-2.6919
NPM1	0.016562	0.569382	-1.6724	0.026498	0.568722	-1.7281
EIF4A1	0.016488	0.569382	-2.3375	0.011535	0.493835	-3.3864
NELFA	0.016392	0.569382	-1.4002	0.015628	0.535589	-2.1152
POLR3C	0.015126	0.569382	-2.6603	0.022907	0.558932	-3.0513
OGT	0.014956	0.569382	-2.3594	0.037516	0.638614	-2.4944
SGF29	0.014332	0.560686	-1.8658	0.0074683	0.403701	-3.4651
LSM10	0.013774	0.548289	-1.3524	0.028366	0.574915	-2.8391
MED11	0.012993	0.54593	-1.7151	0.04513	0.706395	-2.3191
TSG101	0.010654	0.464871	-2.0869	0.032803	0.610493	-2.6885
CHAF1A	0.00814	0.401528	-2.3071	0.006181	0.38855	-3.3811
ACD	0.007573	0.381848	-2.6047	0.0064123	0.38855	-2.6768
NUDT21	0.007481	0.381848	-1.4751	0.027318	0.568722	-2.546
TAF6	0.007159	0.381848	-3.0818	0.0097766	0.450198	-2.225
WDHD1	0.006914	0.381848	-2.3233	0.031695	0.610493	-1.6906
TINF2	0.006705	0.381848	-2.5726	0.00062181	0.235149	-3.8938
DDX54	0.006692	0.381848	-2.1398	0.023155	0.558932	-1.2233
NCBP1	0.006596	0.381848	-2.243	0.0030349	0.363992	-3.4323
SETDB1	0.004933	0.361067	-0.10969	0.037237	0.638614	-2.3892
NKX2-2	0.00485	0.361067	-2.7753	0.047648	0.716379	-1.9971
TAF1	0.004789	0.361067	-2.7003	0.0022669	0.342904	-2.5038
POLR2K	0.00475	0.361067	-3.4833	0.0063948	0.38855	-2.6906
WDR82	0.004697	0.361067	-2.1024	0.0072021	0.403701	-3.4873
SNRPD3	0.002559	0.322607	-2.8719	0.02851	0.574915	-1.5924
CPSF2	0.002097	0.322607	-3.063	0.0066785	0.38855	-2.1015
ELP4	0.001769	0.322607	-3.3659	0.0012153	0.342904	-3.7681
SRSF1	0.001551	0.322607	-3.0208	2.84E-05	0.032178	-3.8401
RAN	0.001521	0.322607	-2.1325	0.049926	0.721807	-2.3094
ING3	0.00115	0.322607	-2.1028	0.0020967	0.342904	-3.0276
TERF2	0.000757	0.322607	-3.5091	0.0098072	0.450198	-4.1737
PSMD14	0.000391	0.322607	-2.8746	0.0019702	0.342904	-3.6126
DHX36	4.58E-05	0.10396	-3.0732	0.0014073	0.342904	-1.9223

Table S4: Biological processes (Gene Ontology) of the essential genes for ORG-63-C dispensable for ORG-63-N (epigenome screen)

This data is available in a separate downloadable Excel file (Supplementary Table S4).

Table S5. All hits for the "druggable" screen.

Hits represented have at least 4 sgRNA out of 6 taken into account for analysis. In bold are represented the hits significant for ORG-63-C (LFC < -1 and p-value < 0,05) but not significant for ORG-63-N (LFC > -1 or p-value > 0,05). Other hits are essential for both cell lines. Fdr= false discovery rate

Gene	p-value_N	fdr_N	lfc_N	p-value_C	fdr_C	lfc_C
**HSP90AA1**	0.70337	0.999997	0.18138	0.02346	0.581416	-1.547
**FANCD2**	0.603	0.999997	-0.22887	0.04947	0.864074	-1.1426
**CDK2**	0.53595	0.999997	-0.27009	0.039806	0.776648	-1.4028
**TOP2B**	0.39481	0.999997	-0.0917	0.0087646	0.38272	-1.5298
**RPTOR**	0.39167	0.999997	-0.45626	0.048055	0.864074	-1.0535
**BCL2L1**	0.31849	0.999997	-0.26447	0.013289	0.459836	-1.2187
**MYBL1**	0.31416	0.999997	-0.5715	0.024879	0.600378	-1.6852
**TTK**	0.28795	0.999997	-0.61574	0.013661	0.463971	-1.584
**FANCC**	0.24945	0.999997	-0.54887	0.0073124	0.38272	-1.9841
**POLE**	0.24504	0.999997	-0.27922	0.013083	0.459836	-1.3574
**CDK12**	0.23879	0.999997	-0.16167	0.008662	0.38272	-1.9851
**GMPS**	0.23684	0.999997	-0.25904	0.035056	0.738989	-1.4074
**CCND3**	0.18021	0.999997	-0.86676	0.017937	0.534732	-1.3585
**USP5**	0.16569	0.999997	-0.87191	0.023271	0.581416	-1.2658
**NFKBIA**	0.14329	0.999997	-0.99465	0.0078846	0.38272	-2.0144
PALB2	0.11743	0.999997	-1.3493	0.018698	0.535814	-1.6932
ERBB2	0.11039	0.987612	-1.4017	0.0062327	0.357209	-1.7004
**PI4KB**	0.10617	0.965347	-0.86682	0.036475	0.741105	-1.4132
CDK6	0.10149	0.945997	-1.1315	0.0024321	0.2478	-1.6764
**FANCE**	0.09315	0.942935	-0.67103	0.026213	0.619383	-1.027
SFPQ	0.091563	0.942822	-1.4153	0.016274	0.514595	-1.7567
CHEK1	0.07284	0.81662	-1.3161	0.037323	0.744027	-1.5696
NFKB1	0.066691	0.794233	-1.4467	0.022871	0.581416	-1.5156
ZRSR2	0.061352	0.762211	-1.3671	0.036152	0.741105	-0.51731
TYMS	0.044762	0.671471	-1.0075	0.0077011	0.38272	-0.54765
ERCC2	0.039321	0.632578	-1.3617	0.0098875	0.40297	-1.8603
PTEN	0.039067	0.632578	-1.2115	0.034791	0.738989	-1.5675
DICER1	0.038684	0.632578	-0.96282	0.044946	0.849801	-1.4766
EGFR	0.035045	0.612117	-1.7131	0.00011067	0.033828	-2.3485
PLK1	0.033776	0.605546	-1.4722	0.029495	0.659684	-1.4826
SRSF2	0.02868	0.559564	-1.6778	0.0032634	0.282853	-2.5253
PSMB1	0.02868	0.559564	-1.6949	0.012614	0.459836	-1.3397
SF1	0.025565	0.54097	-1.7788	0.0070856	0.38272	-1.5811
UGCG	0.024987	0.539138	-1.9558	0.024577	0.600378	-1.6551
MDM4	0.024809	0.539138	-1.2217	0.010805	0.412851	-2.1249
EHMT1	0.023708	0.530246	-2.0165	0.047564	0.864074	-1.411
ATP1A1	0.02218	0.5022	-1.2808	0.0095528	0.402453	-1.2887
MDM2	0.021829	0.5022	-0.64301	0.026342	0.619383	-1.6507
SMC1A	0.021003	0.493844	-1.6252	0.018369	0.534732	-1.8685
FNTA	0.02089	0.493844	-1.7131	0.048784	0.864074	-1.2878
PSMB2	0.020177	0.493844	-1.4236	0.029436	0.659684	-1.2127
INTS4	0.020172	0.493844	-1.8839	0.011898	0.445342	-1.4522
RAD51	0.018989	0.483705	-1.6173	0.036772	0.741105	-1.1482
DDX6	0.017888	0.46867	-1.5258	0.00132	0.186215	-2.42
MTOR	0.017613	0.468145	-1.2723	0.049022	0.864074	-1.2983
BUB1B	0.015767	0.436309	-2.0235	0.014649	0.479756	-1.9845

DHFR	0.014298	0.422948	-1.4787	0.014568	0.479756	-1.7397
KEAP1	0.013591	0.408619	-2.0265	0.0049856	0.315677	-1.4165
PSMD13	0.013008	0.404208	-2.1944	0.016593	0.515774	-1.4073
SF3B1	0.011539	0.371287	-1.197	0.0038681	0.295586	-1.7496
NRAS	0.010784	0.353165	-1.6866	0.021538	0.580212	-1.6319
DDB1	0.0094826	0.316202	-1.4601	0.0031879	0.282853	-1.9743
SMC3	0.0080574	0.284177	-2.0986	0.032756	0.717056	-1.5608
CSNK1A1	0.0073232	0.268614	-1.7082	0.0044349	0.314737	-1.3881
PSMD2	0.0069399	0.265161	-1.6668	0.001849	0.22452	-2.4004
JMJD6	0.0062704	0.24763	-1.9311	0.0036359	0.289927	-2.1337
PTPN11	0.0045429	0.19376	-1.987	0.005018	0.315677	-1.3912
UMPS	0.0045321	0.19376	-1.5241	0.0044619	0.314737	-1.3553
HMGCS1	0.0044295	0.19376	-1.8452	0.001104	0.168729	-2.0009
EIF3G	0.004354	0.19376	-2.1395	0.00062893	0.10486	-1.6842
CCND1	0.0035334	0.17514	-2.1212	0.008716	0.38272	-1.8615
HMGCR	0.0033444	0.17038	-2.3942	0.043445	0.829981	-0.95399
MCL1	0.0030799	0.166133	-2.4753	0.015065	0.484714	-1.7483
RACK1	0.0029881	0.166067	-1.605	0.010222	0.405414	-1.8984
ATR	0.0025022	0.148036	-2.4694	0.00054796	0.100495	-2.0038
SDHD	0.0021459	0.135712	-1.7584	0.003393	0.282853	-2.5881
DNMT1	0.0019786	0.129597	-1.1339	0.028264	0.65616	-1.1749
NPM1	0.0019678	0.129597	-2.0435	0.038646	0.76211	-0.95787
RAD21	0.0016601	0.117098	-2.6024	0.00028343	0.064975	-1.9737
RPL7	0.0013847	0.105817	-2.4844	0.0096554	0.402453	-2.2124
AURKB	0.0013469	0.105817	-2.5235	0.0049694	0.315677	-1.7661
TFDP1	0.0013415	0.105817	-2.9519	0.036297	0.741105	-1.487
ERCC3	0.00087727	0.080446	-2.7568	0.020965	0.580212	-1.7015
CMPK1	0.00048317	0.046639	-2.891	0.023222	0.581416	-1.7001
CDK1	0.00035361	0.036029	-2.3872	0.00014846	0.038897	-2.5161
MED14	0.00028882	0.031159	-2.9184	0.0058386	0.345417	-1.9254
XPO1	0.00021324	0.026073	-3.0787	9.45E-05	0.033828	-3.0555
PTK2	0.00017545	0.022984	-3.2272	0.0020812	0.22452	-2.3706
FASN	0.00016466	0.022984	-3.1806	0.0086188	0.38272	-2.0586
STK11	0.00015926	0.022984	-2.9297	0.013046	0.459836	-1.8787
UBA1	0.00014846	0.022984	-3.2016	2.70E-06	0.002475	-2.7372
RAN	0.00012687	0.022984	-3.1901	0.00042379	0.086359	-2.3079
BRCA1	9.18E-05	0.018702	-2.3419	0.007971	0.38272	-2.0863
RPL10	8.37E-05	0.018702	-2.2912	2.70E-06	0.002475	-3.1212
VHL	8.37E-05	0.018702	-3.1711	0.0018706	0.22452	-2.0348
PSMB5	6.75E-05	0.018702	-2.6779	0.0051637	0.315677	-2.3172
RPL26	6.21E-05	0.018702	-2.8948	2.97E-05	0.013614	-2.8433
AURKA	4.05E-05	0.018564	-2.2227	0.017219	0.52632	-1.992
MYC	2.43E-05	0.014851	-2.9024	2.43E-05	0.013614	-3.3806
TOP2A	2.70E-06	0.002475	-3.851	0.0019786	0.22452	-2.3939
RPL7A	2.70E-06	0.002475	-4.1156	0.0077335	0.38272	-2.1873

Table S6: Biological processes (Gene Ontology) of the essential genes for ORG-63-C dispensable for ORG-63-N (druggable screen)

This data is available in a separate downloadable Excel file (Supplementary Table S6)

Table S7: Details of the CMT and non-neoplastic tissues sent for RNA sequencing

Sample number	Sample name	"D" = Dog number	Diagnosis
1	D1_CMT	1	Carcinoma and malignant myoepithelioma
2	D2_CMT	2	Intraductal papillary carcinoma
3	D3_CMT	3	Carcinoma and malignant myoepithelioma
4	D6_CMT	6	Carcinoma and malignant myoepithelioma
5	D6_nor		Non-neoplastic mammary tissue
6	D8_CMT	8	Comedocarcinoma
7	D10_CMT	10	Carcinoma arising in a complex adenoma
8	D44_nor	44	Non-neoplastic mammary tissue
9	D13_CMT	13	Carcinoma - complex
10	D14_CMT	14	Carcinoma - complex
11	D15_CMT	15	Intraductal papillary carcinoma
12	D15_nor		Non-neoplastic mammary tissue
13	D16_CMT	16	Tubular carcinoma
14	D17_CMT	17	Carcinoma and malign myoepithelioma
15	D17_nor		Non-neoplastic mammary tissue
16	D18_CMT	18	Intraductal papillary carcinoma
17	D18_nor		Non-neoplastic mammary tissue
18	D23_CMT	23	Intraductal papillary carcinoma and ductal carcinoma
19	D46_nor	46	Non-neoplastic mammary tissue
20	D24_CMT	24	Ductal carcinoma
21	D25_1_CMT	25	Ductal carcinoma
22	D25_2_CMT		Complex carcinoma
23	D32_1_CMT	32	Invasive micropapillary carcinoma
24	D32_2_CMT		Tubular carcinoma
25	D32_nor		Non-neoplastic mammary tissue
26	D33_CMT	33	Intraductal papillary carcinoma
27	D49_nor	49	Non-neoplastic mammary tissue
28	D36_CMT	36	Metastasis from a carcinoma
29	D36_nor		Non-neoplastic mammary tissue
30	D41_CMT	41	Carcinoma and malignant myoepithelioma
31	D43_CMT	43	Carcinoma - complex
32	D47_CMT	47	Carcinoma - complex
33	D48_CMT	48	Carcinoma - complex
34	D48_nor		Non-neoplastic mammary tissue
35	D51_CMT_c	51	Carcinoma - complex
36	D51_CMT_s		Solid carcinoma
37	D57_CMT_s	57	Invasive micropapillary carcinoma
38	D57_CMT_c		Carcinoma - complex
39	D57_nor		Non-neoplastic mammary tissue
40	D60_CMT	60	Carcinoma - complex
41	D60_nor		Non-neoplastic mammary tissue
42	D61_CMT	61	Comedocarcinoma
43	D62_nor	62	Non-neoplastic mammary tissue
44	D62_CMT		Carcinoma - complex
45	D63_1_CMT	63	Intraductal papillary carcinoma
46	D63_2_CMT		Complex carcinoma
47	D63_3_CMT		Complex carcinoma

48	D63_nor		Non-neoplastic mammary tissue
49	D20_nor	20	Non-neoplastic mammary tissue
50	D31_nor	31	Non-neoplastic mammary tissue
51	D37_nor	37	Non-neoplastic mammary tissue

Table S8: Differential genes analysis results for the candidates of the CRISPR screens in the RNA sequencing data from CMT (all tumors versus all normal samples)

Epigenetic hits			Druggable hits		
Genes	Log Fold Change	p-value	Genes	Log Fold Change	p-value
SPEN	-0.28963	0.15925	FANCD2	-0.15268	0.732698
VPS72	0.206768	0.116493	**CDK2**	-0.62596	0.009057
HDAC1	-0.14288	0.335846	TOP2B	0.018633	0.929457
ACTL6A	0.018812	0.972947	RPTOR	-0.20426	0.253766
TBL1X	0.063707	0.873384	BCL2L1	-0.10386	0.659623
BAZ1A	-0.35836	0.700323	TTK	-0.1053	NA
TAF8	-0.06698	0.762573	FANCC	0.063731	0.929895
TFDP1	0.086093	0.958588	POLE	-0.48244	NA
CBX5	0.139612	0.555555	CDK12	-0.46169	0.634525
SMARCA5	-0.04029	0.820825	GMPS	0.377255	NA
SRRM1	-0.08818	NA	CCND3	0.434983	0.694774
COPS5	-0.52528	0.392499	USP5	-0.14386	0.509429
NAP1L1	-0.19196	0.797565	**NFKBIA**	-0.75869	0.000137
HASPIN	0.09035	0.870034	PI4KB	0.063198	NA
RCC1	0.164354	0.875178	**FANCE**	-0.29741	0.023788
OIP5	0.089125	0.92375			
SRSF5	-0.11474	NA			
MIS18BP1	-0.46034	NA			
SETD1A	-0.12095	0.382209			
**CDK2**	-0.62596	0.009057			
USP7	-0.14444	0.716551			
**DHX38**	-0.25675	0.00194			
KDM8	-0.2006	NA			
OTUB1	-0.2784	0.321829			
GTF3C4	0.242401	0.475711			
**DDX17**	-0.77266	0.001796			
SNAPC4	-0.24666	0.868034			
MED30	-0.62144	0.570177			
PCNA	0.372448	0.198654			
ECD	-0.32572	0.831708			
UBE2A	-0.00099	0.999582			
GTF2B	0.055338	0.851352			
SMC2	-0.10622	0.830075			
**RUVBL1**	0.398779	0.002099			
AURKB	-0.39973	0.499425			

Genes for which p-value < 0.05 are in bold. NA = non-applicable

Table S9: Differential genes analysis results for the candidates of the CRISPR screens in the RNA sequencing data from organoids (all tumor organoids versus all normal organoids)

Genes for which p-value < 0.05 are in bold.

Epigenetic hits			Druggable hits		
Genes	Log Fold Change	p-value	Genes	Log Fold Change	p-value
VPS72	0.107194971	0.54126	HSP90AA1	-0.073538186	0.67449
PLOD2	0.387992587	0.17589	FANCD2	-0.120815767	0.68104
NCOR1	-0.176984677	0.46221	CDK2	-0.126974043	0.65836
SRSF2	0.116510189	0.57455	TOP2B	-0.157933588	0.37802
HDAC1	-0.012742337	0.95393	RPTOR	-0.139176368	0.5996
ACTL6A	-0.000690347	0.99752	BCL2L1	0.204565159	0.35437
BAZ1A	-0.214563238	0.24268	MYBL1	-0.423681798	0.42349
NOC2L	0.032990096	0.85281	TTK	0.143076475	0.81246
TAF8	-0.012800838	0.96822	FANCC	-0.41518077	0.4173
TFDP1	0.103944967	0.61238	POLE	-0.276456999	0.43674
CBX5	-0.244320086	0.39531	**CDK12**	-0.434281736	0.0407
SMARCA5	-0.294239015	0.09267	GMPS	-0.13859513	0.49784
**SRRM1**	-0.541785653	0.00188	**CCND3**	0.535618147	0.01593
COPS5	0.075613181	0.69185	USP5	0.163576034	0.42251
NAP1L1	-0.182581622	0.559	NFKBIA	0.228511979	0.25245
HASPIN	-0.702098745	0.29611	PI4KB	-0.006445957	0.97652
RCC1	0.070568503	0.72231	FANCE	-0.150349256	0.56219
OIP5	-0.073435752	0.94035			
SRSF5	-0.162102604	0.52346			
MIS18BP1	-0.338844544	0.37022			
RBPMS	0.12985648	0.5605			
SETD1A	-0.061254228	0.78992			
CDK2	-0.126974043	0.65836			
USP7	-0.142383935	0.42685			
SUZ12	-0.513852938	0.06349			
DHX38	-0.009161838	0.96319			
KDM8	0.034953311	0.94186			
OTUB1	-0.290065813	0.52474			
**GTF3C4**	-0.676396374	0.00262			
CENPA	-0.012833882	0.98367			
ZZZ3	-0.263726825	0.22222			
CHEK1	-0.322430303	0.54782			
DDX17	-0.281312539	0.10188			
SNAPC4	0.233555564	0.21631			
MED30	0.036551799	0.8709			
PCNA	0.080238582	0.71112			
ECD	-0.049499269	0.81771			
RSF1	-0.579550258	0.00734			
UBE2A	0.225269293	0.35296			
GTF2B	-0.214405684	0.37257			
SMC2	-0.374398732	0.27185			
**RUVBL1**	0.372779241	0.04911			
AURKB	0.070144206	0.79234			
