# Supplementary material for: Individualized Pooled CRISPR/Cas9 Screenings Identify CDK2 as a Druggable Vulnerability in a Canine Mammary Carcinoma Patient
Source: Vet Sci. 2025 Feb 18;12(2):183. doi: 10.3390/vetsci12020183 (PMC11861728; doi:10.3390/vetsci12020183)
Supplement: Supplementary file 1 [file vetsci-12-00183-s001.zip › Supplementary Figures S1-S4.pdf]

<u>**Supplementary material**</u>

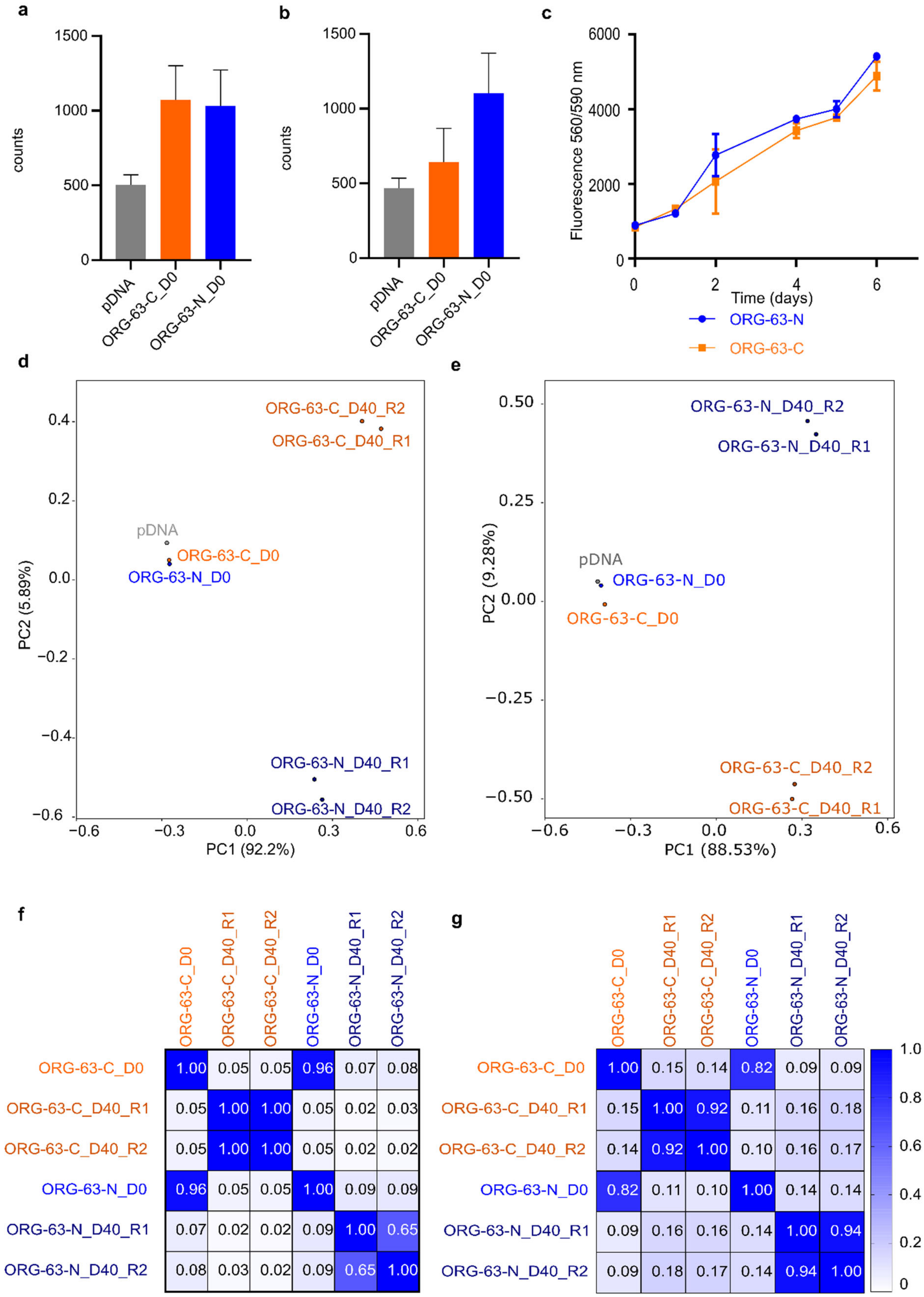

Figure S1: Quality control of the two different pooled CRISPR/Cas9 screens

- **a.** Read counts for the plasmid DNA (pDNA) and day 0 (D0) of the two organoid lines for the CP1736 library ("druggable" library).
- **b.** Read counts for the plasmid DNA (pDNA) and day 0 (D0) of the two organoid lines for the CP1737 library ("epigenome" library).
- **c.** Proliferation assay of tumor (ORG-63-C) and healthy (ORG-63-N) canine mammary organoids. Data represent the mean ± SD of two independent experiments performed in triplicate.
- **d.** Principal component analysis based on the normalized read counts of all samples of the druggable screen (CP1736). Technical replicates are indicated separately.
- **e.** Principal component analysis based on the normalized read counts of all samples of the epigenome screen (CP1737). Technical replicates are indicated separately.
- **f.** Pearson correlation scores based on the normalized read counts of all samples of the druggable screen (CP1736). Technical replicates are indicated separately. Legend is on the **g.** part of the figure.
- **g.** Pearson correlation scores based on the normalized read counts of all samples of the epigenome screen (CP1737). Technical replicates are indicated separately.

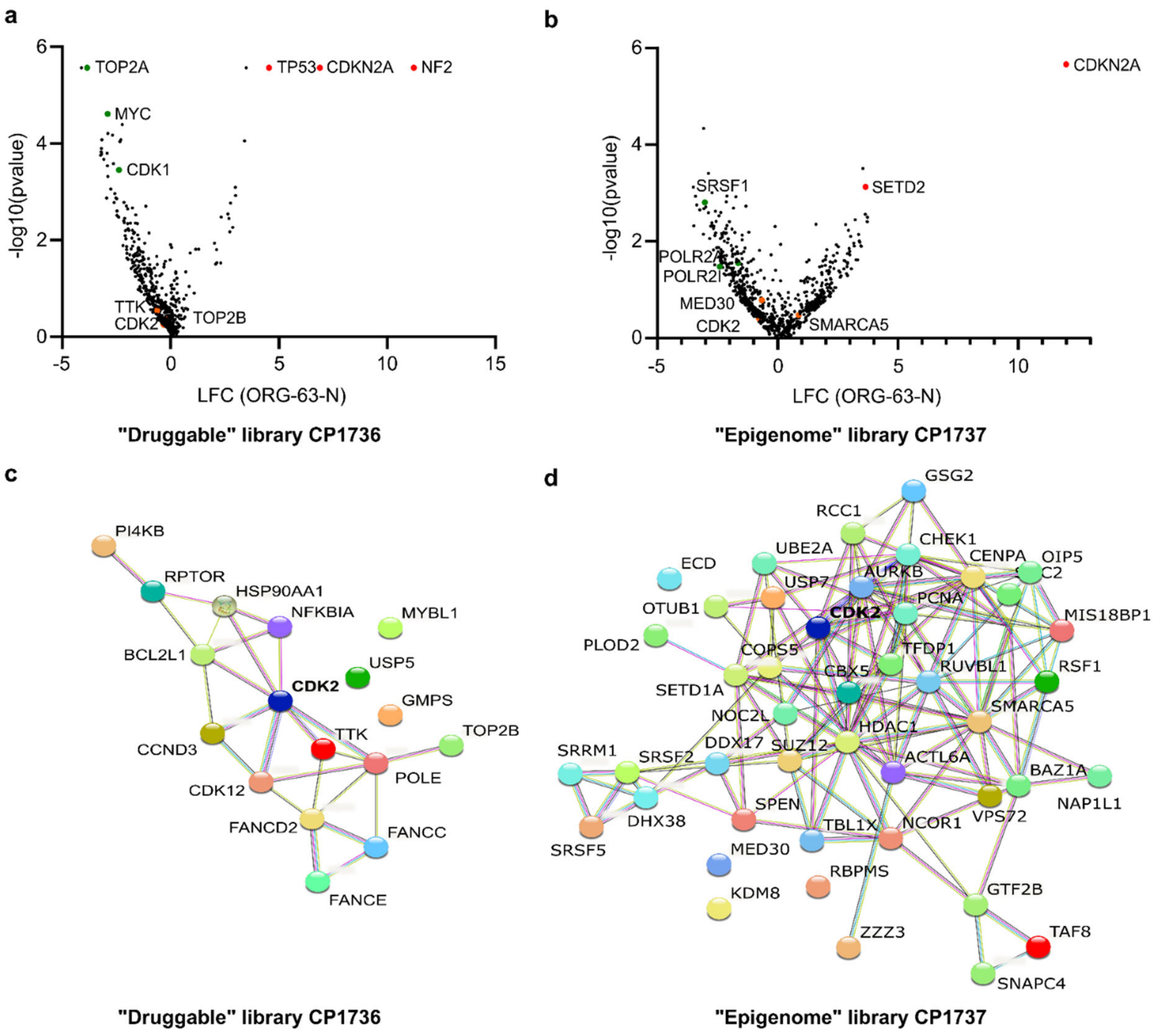

Figure S2: Two independent pooled CRISPR/Cas9 screening approaches in paired CMT organoids to identify therapeutic vulnerabilities of canine mammary carcinoma

- **a.** Volcano plot representing depleted (LFC < 0) and enriched (LFC > 0) genes for ORG-63-N forty days (D40) after D0 for the druggable screen. LFC and p-values were calculated from two technical replicates with MAGeCK analysis. Each dot represents one gene for which at least four sgRNAs (out of 6) were enrolled in the analysis. Selected hits are color-coded.
- **b.** Volcano plot representing depleted (LFC < 0) and enriched (LFC > 0) genes for ORG-63-N forty days (D40) after D0 for the epigenome screen. LFC and p-values were calculated from two technical replicates with MAGeCK analysis. Each dot represents one gene for which at least four sgRNAs (out of 6) were enrolled in the analysis. Selected hits are color-coded.
- **c.** String analysis network for the hits (essential for ORG-63-C but dispensable for ORG-63-N) of the druggable screen.

d. String analysis network for the hits (essential for ORG-63-C but dispensable for ORG-63-N) of the epigenome screen.

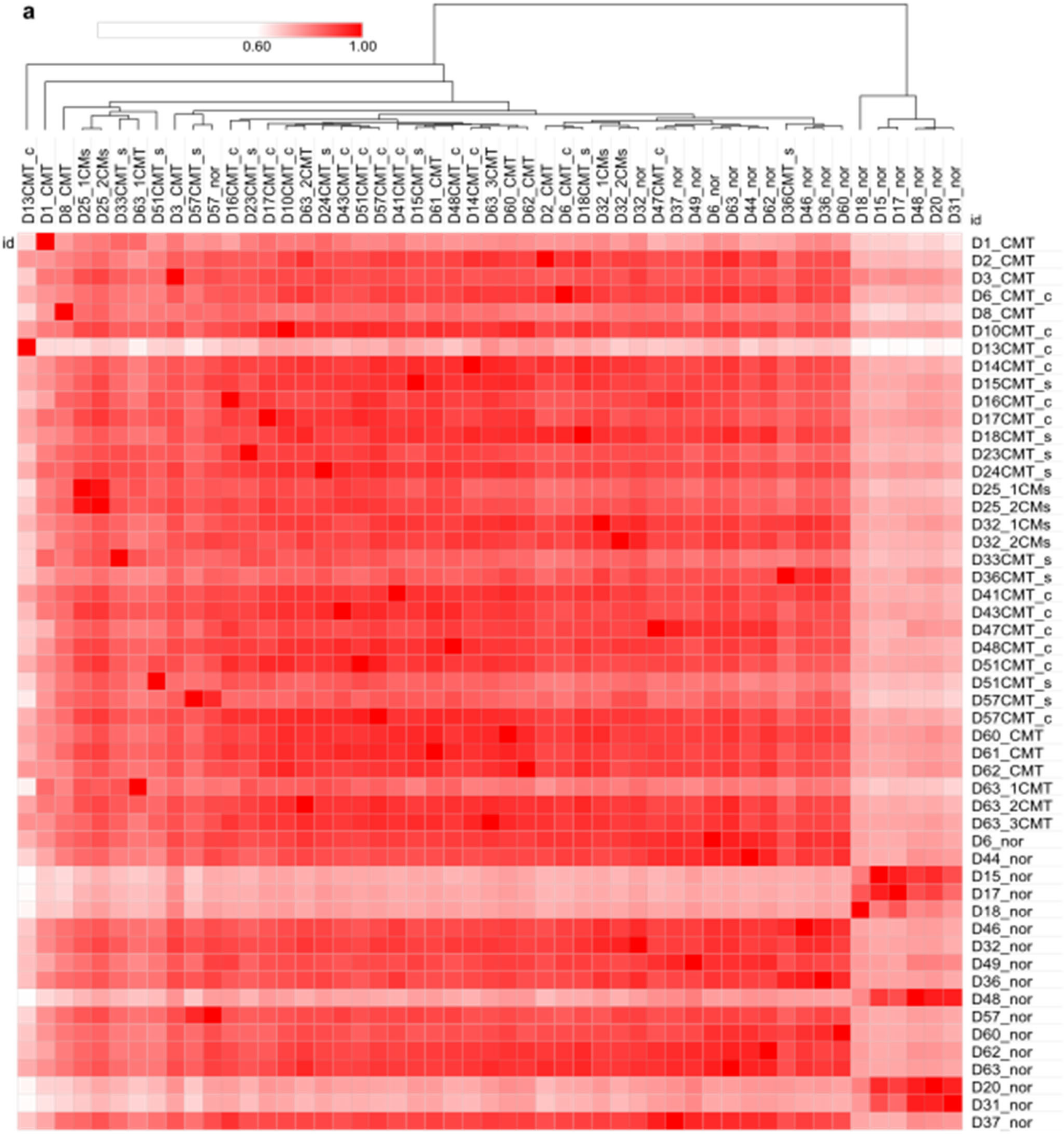

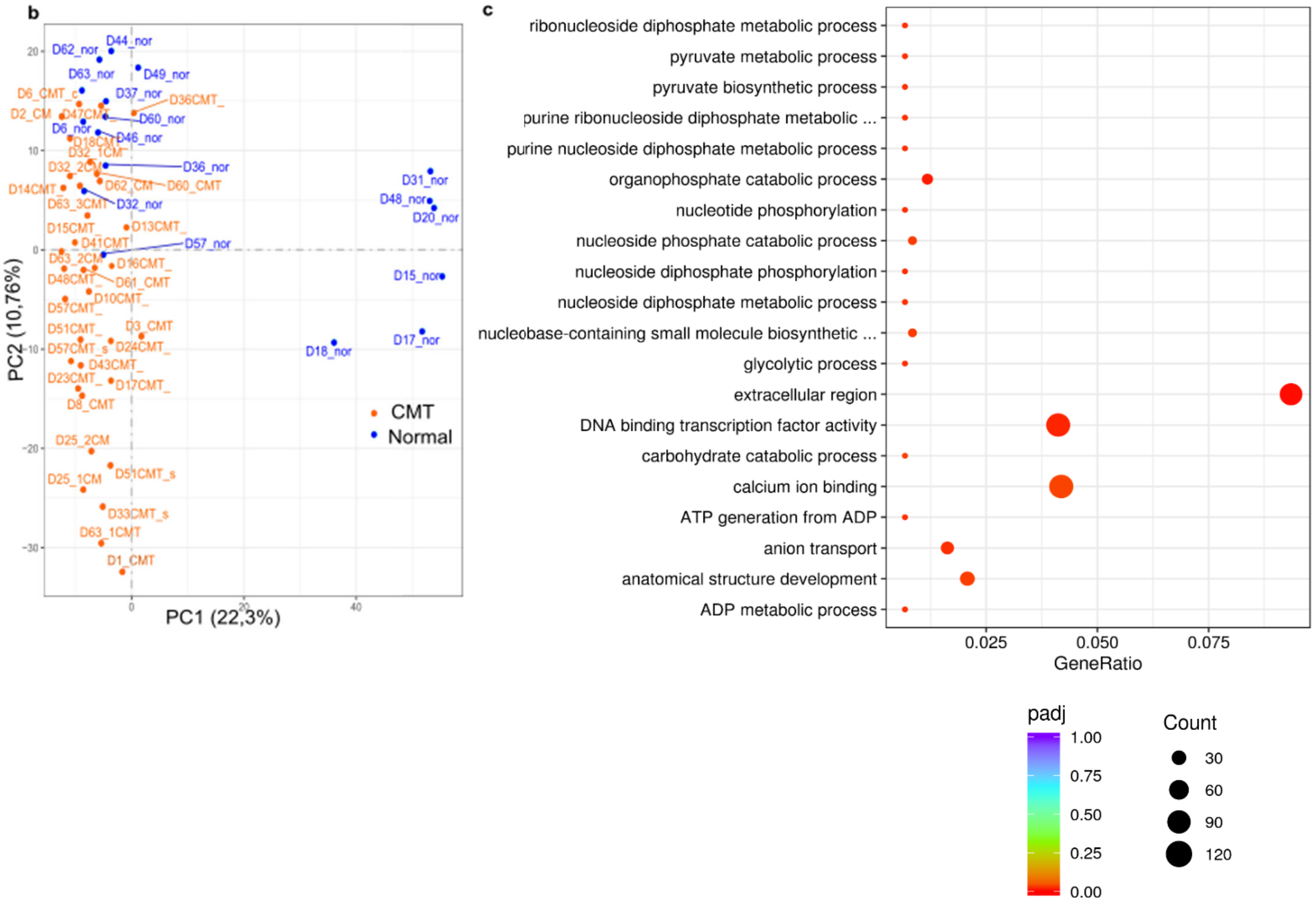

Figure S3: RNA sequencing of CMT and non-neoplastic mammary tissues

- **a.** Unsupervised hierarchical clustering showing the Pearson's correlation heat map generated using normalized FPKM (expected number of Fragments Per Kilobase of transcript sequence per Millions base pairs sequenced of each gene).
- **b.** Principal component analysis of all sequenced samples generated using normalized FPKM (expected number of Fragments Per Kilobase of transcript sequence per Millions base pairs sequenced of each gene).
- **c.** Dot plot showing Gene Ontology analysis results of all differentially expressed genes between CMT and normal mammary tissues. The color of each dot represents the p-value of each term involved in the analysis. The size of each dot represents the gene counts of this term.

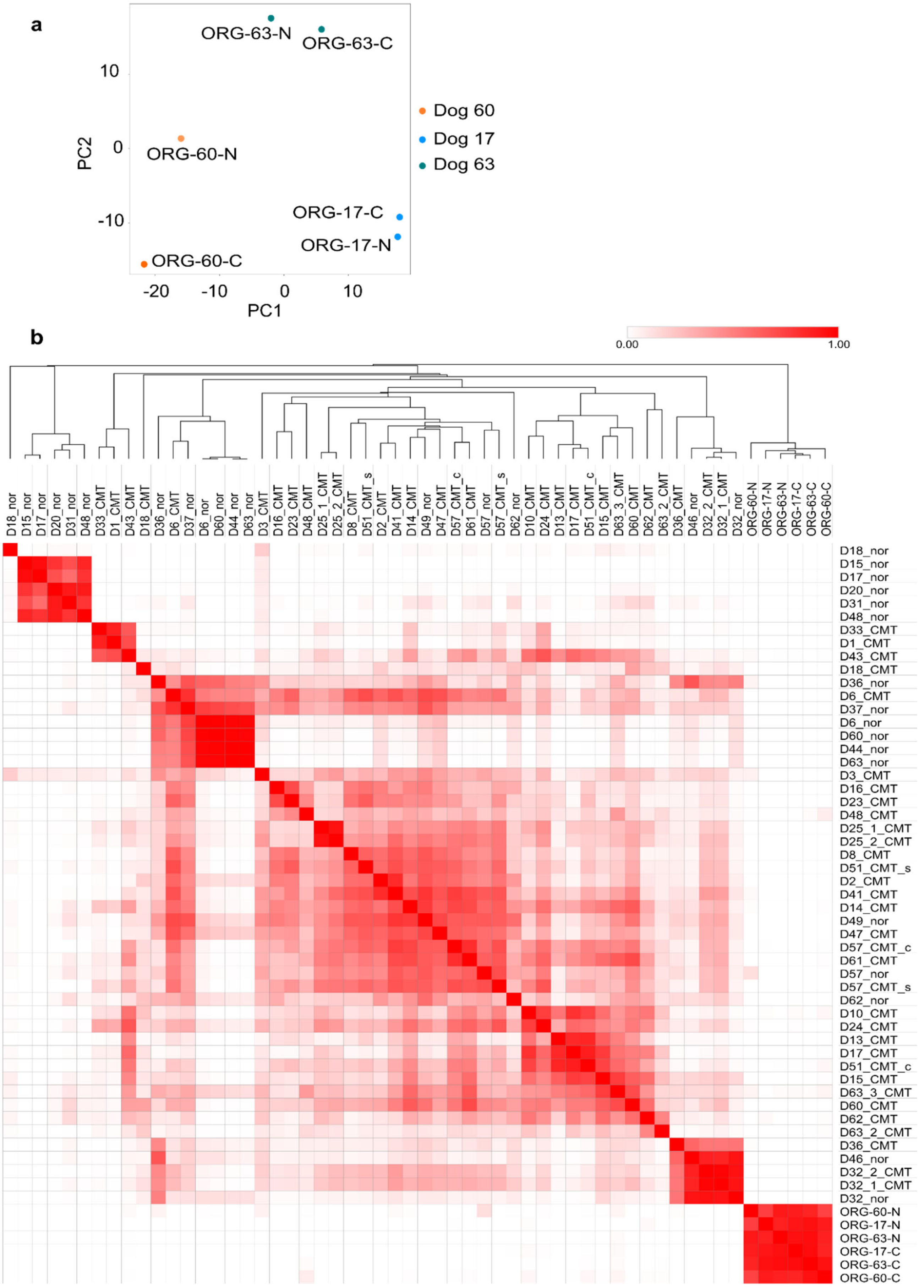
a
ORG-63-N
ORG-63-C
ORG-60-N
ORG-17-C
ORG-17-N
ORG-60-C
Dog 60
Dog 17
Dog 63
PC2
PC1
10
0
-10
-20
b
0.00
1.00
D18_nor
D15_nor
D17_nor
D20_nor
D31_nor
D48_nor
D33_CMT
D1_CMT
D43_CMT
D18_CMT
D36_nor
D6_CMT
D37_nor
D6_nor
D60_nor
D44_nor
D63_nor
D3_CMT
D16_CMT
D23_CMT
D48_CMT
D25_1_CMT
D25_2_CMT
D8_CMT
D51_CMT_s
D2_CMT
D41_CMT
D14_CMT
D49_nor
D47_CMT
D57_CMT_c
D61_CMT
D57_nor
D57_CMT_s
D62_nor
D10_CMT
D24_CMT
D13_CMT
D17_CMT
D51_CMT_c
D15_CMT
D63_3_CMT
D60_CMT
D62_CMT
D63_2_CMT
D36_CMT
D46_nor
D32_2_CMT
D32_1_CMT
D32_nor
ORG-60-N
ORG-17-N
ORG-63-N
ORG-17-C
ORG-63-C
ORG-60-C

Figure S4: RNA sequencing of paired organoids derived from carcinoma and non-neoplastic mammary tissues and comparison to primary tissues

- **a.** Principal component analysis of all organoid samples generated using normalized FPKM (expected number of Fragments Per Kilobase of transcript sequence per Millions base pairs sequenced of each gene).
- **b.** Unsupervised hierarchical clustering of all primary tissues and organoids showing the Pearson's correlation heat map generated using normalized reads per million mapped reads.
