# Supplementary material for: Individualized Pooled CRISPR/Cas9 Screenings Identify CDK2 as a Druggable Vulnerability in a Canine Mammary Carcinoma Patient
Source: Vet Sci. 2025 Feb 18;12(2):183. doi: 10.3390/vetsci12020183 (PMC11861728; doi:10.3390/vetsci12020183)
Supplement: Supplementary file 1 [file vetsci-12-00183-s001.zip › Supplementary Information.pdf]

Supplementary information

Figure S1: Quality control of the two different pooled CRISPR/Cas9 screens

Figure S2: Two independent pooled CRISPR/Cas9 screening approaches in paired CMT organoids to identify therapeutic vulnerabilities of canine mammary carcinoma

Figure S3: RNA sequencing of CMT and non-neoplastic mammary tissues

Figure S4: RNA sequencing of paired organoids derived from carcinoma and non-neoplastic mammary tissues and comparison to primary tissues

Table S1: All 1269 genes targeted by the custom canine CRISPR/Cas9 sub-library CP1737 and sgRNAs counts (plasmid DNA and day 0 and day 40 for both organoid lines)

Table S2: All 834 genes targeted by the custom canine CRISPR/Cas9 sub-library CP1736 and sgRNAs counts (plasmid DNA and day 0 and day 40 for both organoid lines)

Table S3: All hits for the epigenome screen.

Table S4: Biological processes (Gene Ontology) of the essential genes for ORG-63-C dispensable for ORG-63-N (epigenome screen)

Table S5: All hits for the "druggable" screen.

Table S6: Biological processes (Gene Ontology) of the essential genes for ORG-63-C dispensable for ORG-63-N (druggable screen)

Table S7: Details of the CMT and non-neoplastic tissues sent for RNA sequencing

Table S8: Differential genes analysis results for the candidates of the CRISPR screens in the RNA sequencing data from CMT (all tumors versus all normal samples)

Table S9: Differential genes analysis results for the candidates of the CRISPR screens in the RNA sequencing data from organoids (all tumor organoids versus all normal organoids)
